# Deep learning reconstruction for improving the visualization of acute brain infarct on computed tomography

**DOI:** 10.1007/s00234-023-03251-5

**Published:** 2023-11-22

**Authors:** Naomasa Okimoto, Koichiro Yasaka, Nana Fujita, Yusuke Watanabe, Jun Kanzawa, Osamu Abe

**Affiliations:** 1 Department of Radiology, Graduate School of Medicine, The University of Tokyo, 7-3-1 Hongo, Bunkyo-Ku, Tokyo, 113-8655 Japan; 2 Department of Radiology, Tokyo Metropolitan Bokutoh Hospital, 4-23-15 Kotobashi, Sumida-Ku, Tokyo, 130-8575 Japan

**Keywords:** Deep learning, Image processing, Computer-assisted, Brain infarction, Multidetector computed tomography

## Abstract

**Purpose:**

This study aimed to investigate the impact of deep learning reconstruction (DLR) on acute infarct depiction compared with hybrid iterative reconstruction (Hybrid IR).

**Methods:**

This retrospective study included 29 (75.8 ± 13.2 years, 20 males) and 26 (64.4 ± 12.4 years, 18 males) patients with and without acute infarction, respectively. Unenhanced head CT images were reconstructed with DLR and Hybrid IR. In qualitative analyses, three readers evaluated the conspicuity of lesions based on five regions and image quality. A radiologist placed regions of interest on the lateral ventricle, putamen, and white matter in quantitative analyses, and the standard deviation of CT attenuation (i.e., quantitative image noise) was recorded.

**Results:**

Conspicuity of acute infarct in DLR was superior to that in Hybrid IR, and a statistically significant difference was observed for two readers (*p* ≤ 0.038). Conspicuity of acute infarct with time from onset to CT imaging at < 24 h in DLR was significantly improved compared with Hybrid IR for all readers (*p* ≤ 0.020). Image noise in DLR was significantly reduced compared with Hybrid IR with both the qualitative and quantitative analyses (*p* < 0.001 for all).

**Conclusion:**

DLR in head CT helped improve acute infarct depiction, especially those with time from onset to CT imaging at < 24 h.

## Introduction

Acute infarction, which is the primary cause of mortality and disability among the elderly, is characterized by blood clot formation in the brain’s blood vessels or insufficient blood supply to the brain. This causes cerebral tissue ischemia and hypoxia, leading to apoptotic cell death [1]. Two acute infarction treatments have enhanced patient outcomes: tissue plasminogen activator [2] and thrombectomy [3]. However, both treatments are subject to temporal constraints on their use, especially time from onset. CT is more easily accessible and procedurally convenient than MRI. Therefore, CT is widely used at first in routine clinical practice, considering the time. On the other hand, because brain is surrounded by bones, brain CT has been suffered from photon starvation. To alleviate this problem, brain CT examination is usually performed with higher gantry rotation time, high tube current, and sequential scan. However, the contrast-to-noise ratio of acute infarct in CT is still relatively low compared with diffusion-weighted imaging (DWI) despite these inventions, which depict acute infarct as a high-intensity lesion [4].

Deep learning has been garnering significant interest in the field of radiology [5, 6]. It is prominently used not only for lesion detection [7] but also for differential diagnosis [8] and disease staging [9]. Recent studies have demonstrated that deep learning can be effectively applied to image processing [10]. Deep learning reconstruction (DLR) is the particular algorithm. DLR exhibits the capability to improve lesion conspicuity [11] as well as to reduce image noise and enhance image quality in comparison to conventional hybrid iterative reconstruction (Hybrid IR) [12–14]. As for the brain CT, previous studies demonstrated that noise reduction holds promise in enhancing the conspicuity and improving the diagnostic performance of acute infarcts [15, 16]. Therefore, DLR may have a potential to improve the conspicuity of acute infarcts as well as overall image quality. A recent study revealed that DLR reduced noise and improved tissue differentiation [17], but no study has focused on acute infarct lesions with DLR.

This study aimed to compare the conspicuity of acute infarct lesions and image quality on unenhanced head CT between DLR and Hybrid IR.

## Materials and methods

Our Institutional Review Board approved this retrospective study, and the requirement for obtaining written informed consent was waived.

### Patients

**Fig. 1:**
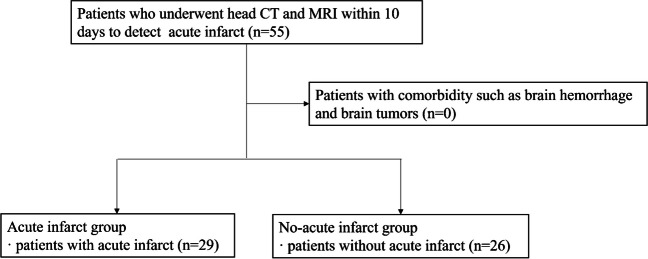
Flow diagram for inclusion of the study

We searched the picture archiving and communication system for all consecutive patients who underwent CT scans for suspected acute infarction from April to September 2022 and those who underwent subsequent MRI with DWI within 10 days (Fig. 1). No patients were excluded during the analysis process.

Patients who underwent unenhanced head CT and MRI with one or more acute infarcts were included in the acute infarct group. The lesions were divided into 5 regions by vascular territory, according to the prior study [18]. A total of 29 patients and 59 acute infarct regions were identified. Two radiologists (A and B with imaging experience of 6 and 13 years, respectively) established the standard for acute infarct diagnosis with reference to MRI.

The inclusion criteria for the no-acute infarct group were all consecutive patients who underwent a CT scan for suspected acute infarct from April to September 2022, and who underwent subsequent MRI with DWI within 10 days. The absence of acute infarct was confirmed based on MRI. Consequently, 26 patients met the criterion.

Imaging parameters for DWI were the following: static magnetic field: 1.5–3.0 T; repetition time/echo time: 4400–6500/59.4–86.0 ms; slice thickness/space between slice: 6.0/6.0–6.5 mm; and acquisition matrix: 128/128–192/192.

The final analyses included 55 patients (29 and 26 patients in the acute infarct and no-acute infarct groups, respectively).

### CT imaging

All patients underwent CT with a multi-detector row CT (Aquilion ONE; Canon Medical Systems, Otawara, Japan). CT scanning parameters were as follows: scan mode: axial; tube voltage: 120 kVp; tube current: automatic tube current modulation with standard deviation set at 2.5; and gantry rotation time: 1.5 s. Images were reconstructed with the following algorithms from the source data: DLR (AiCE BRAIN LCD mild, Canon Medical Systems) and Hybrid IR (AIDR 3D enhanced standard with the kernel of FC64, Canon Medical Systems). The following image reconstruction parameters were similar across all the image sets: field of view: 20–25 cm (adjusted to the head size) and slice thickness/interval: 4/4 mm.

CT images were anonymized and exported from the picture archiving and communication system in Digital Imaging and Communications in Medicine format.

### Qualitative image analyses

Three other radiologists (readers 1, 2, and 3, with 12, 6, and 2 years of imaging experience as radiologists, respectively plus 2 years of imaging experience as interns for all readers) were involved in qualitative image analyses. Qualitative image analyses comprised two parts: lesion depiction (part 1) and image quality (part 2). The three readers evaluated the images using Image J (https://imagej.nih.gov/ij/). In default configuration, window center/window level was set at 25/50 HU, which could be adjusted freely by readers. All the images were randomized by radiologist A before the evaluations by the three readers. Further, the three readers were blinded to the image reconstruction algorithm.

### Part 1: lesion depiction

This part included 29 patients with acute infarcts in 59 regions. Referring to a previous study [18], the brain parenchyma was categorized into the following five regions, with each region further subdivided into left and right hemispheres, resulting in a total of ten regions: anterior cerebral artery (ACA) (A1 and A2); middle cerebral artery at the level of the ventricles above the basal ganglia (sup-MCA) (M4, M5, and M6); middle cerebral artery at level basal ganglia (sub-MCA) (M1, M2, and M3); basal ganglia (BG) (caudate, lentiform nucleus, internal capsule, and insular cortex); and posterior circulation (PCA) (thalamus, superficial PCA, cerebellum, and brainstem).

Initially, the readers were provided with MRI showing the precise acute infarct localization, as indicated by an arrow. The evaluation was performed on the largest lesion in case multiple lesions were present within the same region. Subsequently, the readers evaluated the lesion depiction on CT. The three readers independently evaluated the lesions in terms of lesion depiction with a 5-point scale (5, clear depiction; 4, clearer than standard; 3, standard; 2, blurred than standard; and 1, very blurred).

### Part 2: image quality

This part included 55 patients in the acute infarct or no-acute infarct groups. The three readers, who were blinded to reconstruction algorithms, independently evaluated the image sets in terms of the following:

- Subjective image noise on a 5-point scale (5, almost no noise; 4, less than standard noise; 3, standard noise; 2, more than standard noise; and 1, severe noise)
- Sharpness on a 5-point scale (5, best sharpness; 4, more than standard sharpness; 3, standard sharpness; 2, less than standard sharpness; reduced image quality; and 1, excessive blurring, impairs diagnostic quality)
- Artifacts on a 5-point scale (5, almost no artifact; 4, less than standard artifact; 3, standard artifact; 2, more than standard artifact; and 1, severe artifact)
- Overall image quality on a 5-point scale (5, excellent; 4, better than standard; 3, standard; 2, worse than standard; 1, poor)

### Quantitative image analyses

**Fig. 2:**
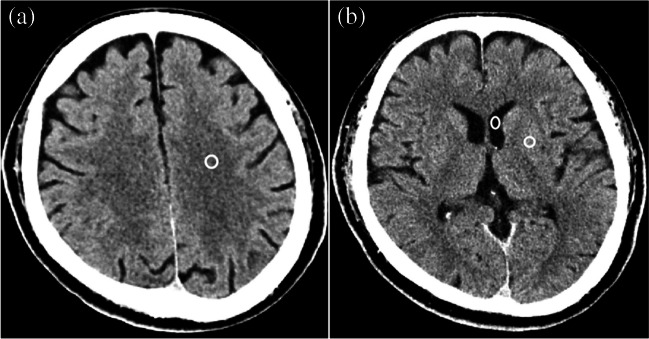
Placing regions of interest (white circles) on (**a**) lateral ventricle and putamen and (**b**) white matter

Radiologist A placed regions of interest with the size of approximately 20 mm^2^ on the lateral ventricle (left anterior horn at the level where the prominence of the caudate nucleus head was most discernible), the putamen where the left putamen was most visible, and white matter of the left convexity (Fig. 2). The apparent lesion was avoided in placing regions of interest on these normal structures. Regions of interest were also placed on acute infarct. The standard deviation (SD) of the CT attenuation for normal structures, which is an indicator of quantitative image noise, was recorded. The CT attenuation of white matter and acute infarct was also recorded, and the contrast between them (i.e., absolute value for the difference of them) was calculated. In addition, the contrast-to-noise ratio (CNR) was calculated with the contrast divided by image noise in the lateral ventricle. These evaluations were performed with Image J (https://imagej.nih.gov/ij/).

In addition, to evaluate the optimal window setting for each reconstruction algorithm, radiologist B adjusted the window width and window center for each image set. During this evaluation, window setting was concealed. After adjusting window setting for all the patients, the adjusted window width and window center was recorded.

### Statistical analysis

EZR version 1.55 (https://www.jichi.ac.jp/saitama-sct/SaitamaHP.files/statmed.html) [19], which is a graphical user interface of R version 4.1.2 (https://www.r-project.org/) (R Foundation for Statistical Computing, Vienna, Austria), was used for statistical analyses.

Fisher’s exact test and the Mann–Whitney U test were used to compare the demographic and clinical characteristics between the acute infarct and no-acute infarct groups. The paired t-test and Wilcoxon signed-rank test compared the results for continuous variables and ordinal scales between DLR and Hybrid IR, respectively. A *p-*value of < 0.05 was considered statistically significant for these comparisons. Subgroup analyses in terms of region, size (≥ 10 mm or < 10 mm), and time from onset to CT (< 4.5 h and < 24 h) were also performed in lesion conspicuity evaluations.

Cohen’s kappa analysis evaluated interobserver agreement. The kappa value of 0.00–0.20, 0.20–0.40, 0.41–0.60, 0.61–0.80, and 0.81–1.00 indicate poor, fair, moderate, good, and excellent agreement, respectively.

## Results

### Patients

Table 1 described detailed patient background information. Acute infarct and no-acute infarct groups consisted of 29 (mean age, 75.8 ± 13.2 years; 20 males) and 26 (mean age, 64.4 ± 12.4; 18 males) patients, respectively. Statistically significant differences were found in age (*p* < 0.001) and the presence of hypertension (*p* = 0.031) between the acute infarct and no-acute infarct groups. No patient started treatment with tissue plasminogen activator or thrombus retrieval therapy between CT and MRI.

**Table 1:** Patient background information

	Acute infarct	No-acute infarct	*P*-value
Number of patients	29	26	
Age (years: mean ± standard deviation)	75.8 ± 13.2	64.4 ± 12.4	< 0.001 ^a^
Sex (male, female)	20, 9	18, 8	1.000 ^b^
History of cerebral infarct (positive, negative)	5, 24	2, 24	0.426 ^b^
Hypertension (positive, negative)	18, 11	8, 18	0.031 ^b^
Hyperlipidemia (positive, negative)	7, 22	5, 21	0.751 ^b^
Diabetes mellitus (positive, negative)	9, 20	4, 22	0.215 ^b^

^a^ Mann–Whitney U test

^b^ Fisher’s exact test

**Fig. 3:**
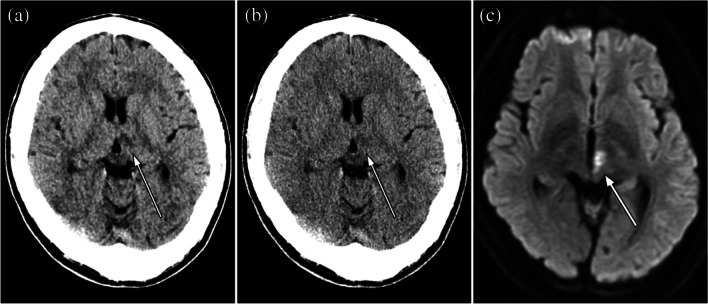
Unenhanced head CT (**a**, **b**) of a 37-year-old male patient with acute infarct in the left posterior circulation region (white arrows). The time interval from onset to CT examination was 5 h. The conspicuity of this acute infarct was rated as 5, 3, and 1 in DLR (**a**) and 3, 2, and 2 in Hybrid IR (**b**) by readers 1, 2, and 3, respectively. Window level / width is 25 / 50 Hounsifield unit for both (**a**) and (**b**). Diffusion-weighted image is also shown in (**c**)

**Fig. 4:**
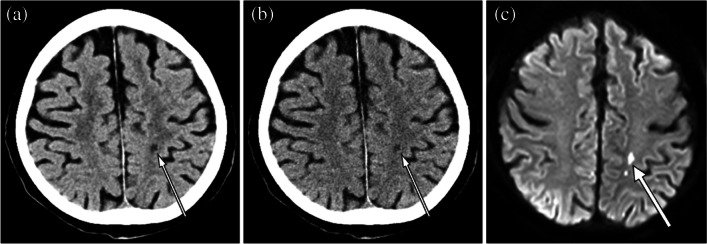
Unenhanced head CT (**a**, **b**) of a 65-year-old female patient with acute infarct in the left sup-MCA region (white arrows). The time interval from onset to CT examination was 9 h. The conspicuity of this acute infarct was rated as 5, 4, and 5 in DLR (**a**) and 4, 3, and 3 in Hybrid IR (**b**) by readers 1, 2, and 3, respectively. Window level / width is 25 / 50 Hounsifield unit for both (**a**) and (**b**). Diffusion-weighted image is also shown in (**c**)

**Fig. 5:**
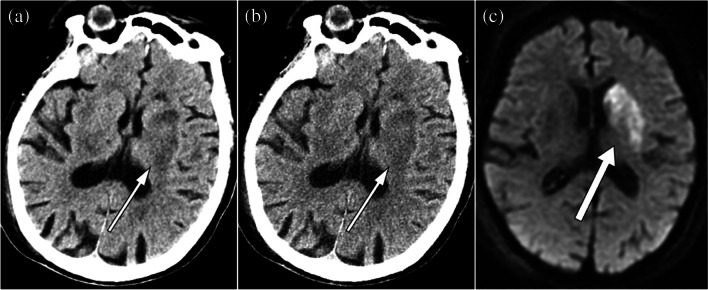
Unenhanced head CT (**a**, **b**) of an 85-year-old female patient with acute infarct in the left basal ganglia region (white arrows). The time interval from onset to CT examination was 9 h. The conspicuity of this acute infarct was rated as 3, 3, and 4 in DLR (**a**) and 2, 3, and 2 in Hybrid IR (**b**) by readers 1, and 2, and 3 respectively. Window level / width is 25 / 50 Hounsfield unit for both (**a**) and (**b**). Diffusion-weighted image is also shown in (**c**)

The number and percentage of acute infarct positive regions were 0/18/5/12/24 and 0.0%/30.5%/8.5%/20.3%/40.7%, respectively for ACA/sup-MCA/sub-MCA/BG/PCA region in the acute infarct group. The number of regions with acute infarct diameters of < 10 mm or ≥ 10 mm was 21/38 and those with acute infarct time of < 4.5 h/ < 24 h from onset to CT were 16/46. Representative CT images are shown in Figs. 3, 4, and 5.

### Qualitative image analyses (part 1): lesion conspicuity

The detailed results of the qualitative image analyses are shown in Table 2. All lesion depictions in DLR tended to be superior to those in Hybrid IR for all readers, and statistically significant differences were observed for readers 1 and 3 (*p* ≤ 0.038).

**Table 2:** Depiction of acute infarct lesion

	Reader 1			Reader 2			Reader 3		
	DLR	Hybrid IR	*P*-values	DLR	Hybrid IR	*P*-values	DLR	Hybrid IR	*P*-values
Area									
ACA	0/0/0/0/0	0/0/0/0/0	N/A	0/0/0/0/0	0/0/0/0/0	N/A	0/0/0/0/0	0/0/0/0/0	N/A
Sup-MCA	5/1/2/5/5	0/6/1/4/7	0.037	0/4/3/3/8	0/2/4/4/8	0.608	6/0/3/3/6	4/0/3/4/7	0.341
Sub-MCA	1/1/0/1/2	1/0/1/3/0	0.850	0/1/0/1/3	0/1/1/0/3	1.000	2/0/1/1/1	1/0/1/1/2	0.371
BG	1/1/2/3/5	1/1/1/5/4	1.000	0/1/1/3/7	0/1/3/2/6	0.345	1/3/2/5/1	1/1/2/3/5	0.040
PCA	6/2/5/4/7	2/3/4/7/8	0.006	0/5/5/9/5	0/3/5/5/11	0.008	6/1/2/3/12	4/2/2/6/10	0.718
Size									
< 10 mm	2/1/2/6/10	0/1/2/7/11	0.080	0/2/0/5/14	0/0/2/4/15	0.233	4/2/4/1/10	2/0/3/6/10	0.140
≥ 10 mm	11/4/7/7/9	4/9/5/12/8	0.029	0/9/9/11/9	0/7/11/7/13	0.318	11/2/4/11/10	8/3/5/8/14	0.108
Time									
< 4.5 h	2/0/2/3/9	1/0/3/6/6	0.824	0/0/1/7/8	0/0/1/2/13	0.073	2/1/0/6/7	0/1/3/3/9	0.392
< 24 h	9/1/8/9/19	1/6/6/17/16	0.020	0/8/7/14/17	0/2/11/8/25	0.004	9/3/5/12/17	3/2/8/10/23	0.014
All	13/5/9/13/19	4/10/7/19/19	0.005	0/11/9/16/23	0/7/13/11/28	0.157	15/4/8/12/20	10/3/8/14/24	0.038

The number of patients for each score (5/4/3/2/1) is shown

*ACA* anterior cerebral artery; *BG* basal ganglia; *DLR* deep learning reconstruction; *Hybrid IR* hybrid iterative; *N/A* not applicable; *PCA* posterior circulation; *sub-MCA* middle cerebral artery at level basal ganglia: and *sup-MCA* middle cerebral artery at the level of the ventricles above the basal ganglia

Comparisons were performed with the Wilcoxon signed-rank test

DLR improved the acute infarct depiction compared with Hybrid IR in the PCA region for readers 1 and 2 (*p* ≤ 0.008), in sup-MCA region for reader 1 (*p* = 0.037), and in BG region for reader 3 (*p* = 0.040) in the subgroup analysis. DLR significantly improved the acute infarct depiction with onset to CT imaging time at < 24 h compared with Hybrid IR for all readers (*p* ≤ 0.020). Conspicuity for acute infarct with onset to CT imaging time at < 4.5 h in DLR tended to improve for readers 2 and 3. Conspicuity of acute infarct with size ≥ 10 mm in DLR was superior to that in Hybrid IR, and a statistically significant difference could be observed for one reader (*p* = 0.029).

The interobserver agreement in lesion conspicuity evaluations was moderate (kappa = 0.560) between reader 1 and 2, good (kappa = 0.784) between reader 1 and 3, and moderate (kappa = 0.570) between reader 2 and 3.

### Qualitative image analyses (part 2): image quality

Table 3 shows the detailed results of the qualitative image quality analyses. All readers agreed that DLR was significantly superior to Hybrid IR in terms of noise (*p* < 0.001). Conversely, controversial results were observed for readers 1 and 3 in terms of sharpness; reader 1 rated Hybrid IR was significantly superior to DLR (*p* < 0.001) and reader 3 rated vice versa *(p* = 0.004). Overall image quality in DLR was rated as superior to that in Hybrid IR by readers 2 and 3 (*p* ≤ 0.043).

**Table 3:** Results for qualitative image analyses

	Reader 1			Reader 2			Reader 3		
	DLR	Hybrid IR	*P*-values	DLR	Hybrid IR	*P*-values	DLR	Hybrid IR	*P*-values
Noise	2/37/16/0/0	0/3/25/26/1	< 0.001	0/21/30/4/0	0/5/38/12/0	< 0.001	3/44/6/2/0	0/2/23/29/1	< 0.001
Sharpness	0/0/33/22/0	0/0/53/2/0	< 0.001	0/13/40/2/0	0/16/36/3/0	0.694	0/22/16/17/0	0/4/29/22/0	0.004
Artifact	0/12/33/10/0	0/16/25/13/1	0.848	0/13/37/5/0	0/6/41/8/0	0.008	1/23/26/5/0	0/3/39/12/1	< 0.001
Overall	0/1/43/11/0	0/0/51/4/0	0.117	0/22/30/3/0	0/15/32/8/0	0.043	0/31/12/12/0	0/2/28/24/1	< 0.001

The numbers of patients for each score (5/4/3/2/1) are shown

*DLR* deep learning reconstruction; *Hybrid IR* hybrid iterative reconstruction

Comparisons were performed with the Wilcoxon signed-rank test

The interobserver agreement in the evaluations of noise, sharpness, artifact, and overall was moderate (kappa = 0.418), fair (kappa = 0.354), moderate (kappa = 0.478), and fair (kappa = 0.381) between reader 1 and 2, good (kappa = 0.668), poor (kappa = 0.124), fair (kappa = 0.356), and poor (kappa = 0. 099) between reader 1 and 3, and moderate (kappa = 0.475), poor (kappa = 0.188), moderate (kappa = 0.458), and fair (kappa = 0. 343) between reader 2 and 3, respectively.

### Quantitative image analyses

Table 4 shows detailed results for the quantitative image analyses. The quantitative image noises (mean ± SD) were statistically significantly reduced in DLR than those in Hybrid IR for all structures (*p* < 0.001 for all). The contrast between acute infarct and white matter in DLR (13.5 Hounsfield unit [HU]) was significantly higher than that in Hybrid IR (9.9 HU) (*p* < 0.001). CT attenuation of the lesion/white matter was 23.7/37.2 HU and 23.3/31.8 HU in DLR and Hybrid IR, respectively. There was also statistically significant difference in CNR between DLR (6.5) and Hybrid IR (3.0) (*p* < 0.001).

**Table 4:** Results for quantitative image analyses

		DLR	Hybrid IR	*P*-values
Image noise	Lateral ventricle	2.76 ± 0.49	3.15 ± 0.52	< 0.001
	Putamen	2.45 ± 0.76	3.34 ± 0.88	< 0.001
	White matter	2.23 ± 0.49	3.38 ± 0.61	< 0.001
Contrast		13.5 ± 7.3	9.9 ± 5.0	< 0.001
CNR		6.5 ± 3.6	3.0 ± 1.8	< 0.001

*CNR* contrast-to-noise ratio; *DLR* deep learning reconstruction; *Hybrid IR* hybrid iterative reconstruction

Contrast was the difference of the CT attenuation between lesion and white matter

Contrast-to-noise ratio was calculated with contrast divided by image noise in lateral ventricle

Comparisons were performed with the paired *t*-test

The optimal window width was 64.2 ± 8.2 HU and 60.8 ± 9.5 HU for DLR and Hybrid IR, respectively. There was no statistically significant difference between them (*p* = 0.095). The optimal window center in DLR was 38.8 ± 2.1 HU, which was significantly higher than that in Hybrid IR (32.8 ± 2.7 HU) (*p* < 0.001).

## Discussion

Head CT is more readily accessible and is associated with shorter examination time compared with MRI. This study revealed that DLR significantly decrease image noise compared with Hybrid IR on unenhanced head CT, which improved the conspicuity of acute infarct, especially for those with time from onset to CT examination of < 24 h.

Several studies reported that DLR helps improve image quality compared with Hybrid IR in head CT [17, 20, 21]. The association between the degree of image noise *vs.* the visibility and diagnostic performance of acute infarction on CT has been indicated by previous studies which used image filters or iterative reconstruction [15, 16]. However, no study has directly investigated DLR’s potential in improving image quality results in superior lesion depiction. Our study revealed that DLR effectively reduced image noise in both the qualitative and quantitative image analyses (*p* < 0.001 for all). This would have been resulted in improved CNR for the acute infarct when compared to Hybrid IR in head CT (*p* < 0.001). The acute infarct conspicuity in DLR also tended to be superior to that in Hybrid IR for all readers and statistically significant difference could be observed for two readers (*p* ≤ 0.038).

Our study is unique because we evaluated the effects of DLR on acute infarct depiction based on region, size, and time from onset to CT. This study revealed that DLR improved acute infarct depiction in the PCA region for two readers and the sup-MCA and BG regions in one reader. Further, the incidence of acute infarct in these regions is relatively high (30.5%/40.7% for sup-MCA/PCA), which is compatible with a previous study (29.9%/27.4% for sup-MCA/PCA) [18]. Hence, DLR might have a potential to improve acute infarct depiction in frequent sites. Additionally, our study revealed that acute infarct depiction with ≥ 10 mm was improved for one reader. Notably, acute infarct conspicuity with time from onset to CT imaging of < 24 h, which is a candidate for thrombus retrieval therapy [3], was significantly improved. DLR may help compare and interpret the follow-up CT examination with the initial CT for these patients.

However, DLR-associated drawback was observed. In general, sharpness is known to have negatively associated with the degree of noise [22]. Our study judged image sharpness in DLR as significantly deteriorated by one reader (*p* < 0.001). On the other hand, overall image quality was evaluated by considering not only image noise but also sharpness and artifacts. Therefore, while image noise in DLR was judged as significantly superior to that in Hybrid IR, significant superiority of overall image quality in DLR as compared to Hybrid IR was observed only for two readers. How this affects the depiction of brain diseases other than acute infarct needs to be investigated in future research.

Contrast between lesion to white matter in DLR was found to be significantly higher than that in Hybrid IR. As for the CT attenuation of structures in various reconstruction algorithms, there have been mixed results. According to a systematic review, CT attenuation of abdominal CT images was similar between DLR, Hybrid IR, and filtered back projection [14]. However, there also exist reports which reported the difference of CT attenuation of the liver between model-based iterative reconstruction and filtered back projection [23, 24]. In addition, Yamakuni, et al. recently reported that the CT attenuation of the cerebral venous sinus in DLR was significantly higher than that in Hybrid IR [25]; our results would be in line with their article.

This study has some limitations. First, a lesion detection test was not performed in this study because a comparison with previous CT examinations would be necessary for diagnosing acute infarcts in some patients with chronic ischemic change or old infarcts. Instead, we aimed to show the superiority of DLR in terms of acute infarct conspicuity. Second, this study included a relatively small number of participants, which would have caused various statistical test results across readers in the subgroup analyses. Further studies that include a larger number of patients would be warranted while statistically significant differences in lesion depiction could be observed between DLR and Hybrid IR for some readers. Third, the results for the depiction of acute infarction lesion, sharpness, artifact, and overall were different between the readers, because the readers exhibit varying levels of experience and familiarity with the images, possibly stemming from differences in their years of experience. Furthermore, as mentioned in the limitations, small number of the lesions may also be a contributing factor. Fourth, window setting used in the qualitative image analyses was not recorded. Instead, we evaluated the optimal window in the quantitative image analyses and significant difference was observed for optimal window center between DLR and Hybrid IR. However, we assume the readers evaluated images with appropriate window setting because they were allowed to adjust window center and window width in evaluating images. Fifth, there were multiple lesions for some patients. Instead of selecting one lesion for each patient, which could lead to bias, we selected to analyze the conspicuity of lesions for ten territories based on a previous article by van Ommen, et al. [18]. Sixth, there were some patients with a relatively long period between CT and MRI examinations, up to 10 days. Thereby, there could be some degree of changes in images. Finally, each manufacturer’s DLR has subtle differences in algorithms; thus, the study results are not necessarily applicable to the DLR of other manufacturers.

In conclusion, DLR significantly reduced image noise compared with Hybrid IR, thereby improving acute infarct depiction, especially for those with time from onset to CT examination of < 24 h.
